# Prophylactic Effect of Probiotics on the Development of Experimental Autoimmune Myasthenia Gravis

**DOI:** 10.1371/journal.pone.0052119

**Published:** 2012-12-20

**Authors:** Chang-Suk Chae, Ho-Keun Kwon, Ji-Sun Hwang, Jung-Eun Kim, Sin-Hyeog Im

**Affiliations:** School of Life Sciences and Immune Synapse Research Center, Gwangju Institute of Science and Technology (GIST), Buk-gu, Gwangju, Korea; University Hospital of Heidelberg, Germany

## Abstract

Probiotics are live bacteria that confer health benefits to the host physiology. Although protective role of probiotics have been reported in diverse diseases, no information is available whether probiotics can modulate neuromuscular immune disorders. We have recently demonstrated that IRT5 probiotics, a mixture of 5 probiotics, could suppress diverse experimental disorders in mice model. In this study we further investigated whether IRT5 probiotics could modulate the progression of experimental autoimmune myasthenia gravis (EAMG). Myasthenia gravis (MG) is a T cell dependent antibody mediated autoimmune disorder in which acetylcholine receptor (AChR) at the neuromuscular junction is the major auto-antigen. Oral administration of IRT5 probiotics significantly reduced clinical symptoms of EAMG such as weight loss, body trembling and grip strength. Prophylactic effect of IRT5 probiotics on EMAG is mediated by down-regulation of effector function of AChR-reactive T cells and B cells. Administration of IRT5 probiotics decreased AChR-reactive lymphocyte proliferation, anti-AChR reactive IgG levels and inflammatory cytokine levels such as IFN-γ, TNF-α, IL-6 and IL-17. Down-regulation of inflammatory mediators in AChR-reactive lymphocytes by IRT5 probiotics is mediated by the generation of regulatory dendritic cells (rDCs) that express increased levels of IL-10, TGF-β, arginase 1 and aldh1a2. Furthermore, DCs isolated from IRT5 probiotics-fed group effectively converted CD4^+^ T cells into CD4^+^Foxp3^+^ regulatory T cells compared with control DCs. Our data suggest that IRT5 probiotics could be applicable to modulate antibody mediated autoimmune diseases including myasthenia gravis.

## Introduction

Probiotics are live microorganisms which provide beneficial effects on the host organism by competing with potential pathogen and by producing antibacterial agents known as bacteriocins [1]. Health promoting effects of probiotics are also associated with modulation of immune responses. For example, probiotic treatment augments the secretion of IgA in the gut and reduces the pathogens [2]. Recent studies have shown the strain-specific beneficial effect of probiotics in modulating diverse immune disorders. For example, lactobacilli and bifidobacterium showed therapeutic effects in rheumatoid arthritis, inflammatory bowel disease and atopic dermatitis [3], [4], [5], [6], [7], [8]. These therapeutic effects of probiotics are achieved through regulation of cytokine expression and modulation of DC function [9], [10]. Single strain of probiotics or a mixture of several kinds of probiotics is effective for maintaining the homeostasis of immune system in the inflammatory diseases [11], [12]. Recently, we have demonstrated that IRT5 probiotics, a mixture of 5 probiotics, could suppress diverse immune disorders through generation of CD4^+^Foxp3^+^ Tregs in mouse model [13].

Myasthenia gravis (MG) is a systemic autoimmune disease caused by autoantibodies specific for nicotinic acetylcholine receptor (AChR) at the neuromuscular junction (NMJ). Binding of auto-antibodies to AChR results in the failure of NMJ. Experimental autoimmune myasthenia gravis (EAMG) has been extensively studied to elucidate the pathogenic mechanism. Serum from MG patients or anti-AChR antibodies from EMAG developed the MG symptoms when transferred to the rodents [14]. Removal of anti-AChR antibodies reduces the severity of MG symptoms [15], [16]. MG patients have abundant anti-AChR reactive T helper cells that mainly produce pro-inflammatory cytokines such INF-γ and TNF-α [17], [18], [19]. Although many facts of MG pathogenesis were elucidated, proper remedies for treating MG are not fully developed.

In the present study, we investigated whether IRT5 probiotics have protective effects on the development of EAMG. The probiotics mixture (IRT5) consists of a combination of five probiotic strains in equal ratio; *Streptococcus thermophilus* (ST), *Lactobacillus reuteri* (LR), *Bifidobacterium bifidium* (BB), *Latobacillus acidophilus* (LA) and *Latobacillus casei* (LC). Previously, we have demonstrated that oral administration of IRT5 probiotics showed therapeutic effects on experimental disease models such as TNBS-induced inflammatory bowel disease (IBD) and atopic dermatitis (AD) [13]. Based on this fact, we further questioned whether IRT5 treatment could also modulate EAMG in rats which mimic the long-lasting chronic disease of human MG. The prophylactic effect of IRT5 probiotics administration against EAMG development was examined by measuring clinical score, complement deposition, anti-AChR antibody levels, pro-inflammatory cytokine levels and AChR-reactive lymphocytes proliferation. Oral administration of IRT5 probiotics suppressed AChR-reactive pro-inflammatory lymphocyte response by inducing regulatory dendritic cells (rDCs) that effectively converted CD4^+^ T cells into CD4^+^Foxp3^+^ regulatory T cells.

## Results

### Oral Administration of Probiotics Prevents EAMG Development

**Figure 1:**
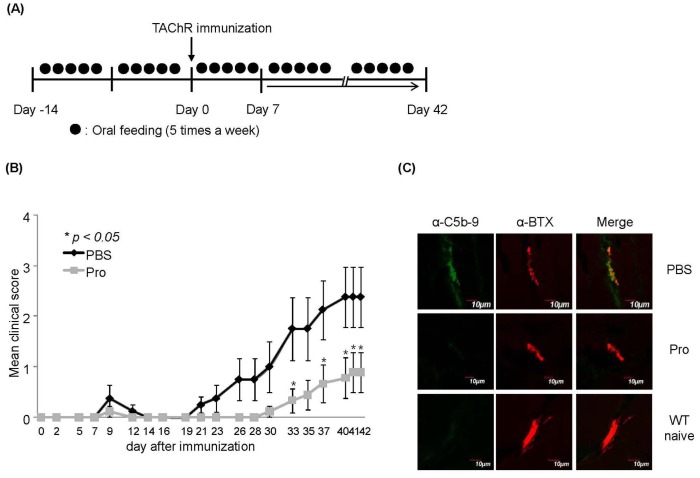
Oral administration of IRT5 probiotics suppresses EAMG progression. Oral administration of IRT5 probiotics or PBS as a control was initiated two weeks before immunization of TAChR and continued till the end of experiment (6 weeks after induction of EAMG) (A). Effect of IRT5 probiotics or PBS treatment on EAMG progression was analyzed by monitoring clinical score every other day (B). Mean clinical score was evaluated based on the standard clinical score criteria. The points and bars represent means and standard deviations, respectively. Data are representative of three independent experiments. *p<0.05. Complement deposition (C) and weight change (Table 1) of each treatment group were assessed. To assess the presence of AChR and complement at the NMJ in each treatment group, 10–15 muscle sections were analyzed (AChR: red fluorescence, complement C5b-9: green fluorescence). Result shown is one representative section. Normal non-immunized rats were employed as healthy control (positively stained for AChR but negative for C5b-9).

The main purpose of this study is to evaluate the prophylactic effect of IRT5 probiotics against EAMG development and elucidate the underlying mechanism of immunoregulatory function. Rats were orally administered either with control PBS or PBS containing IRT5 probiotics five times per weeks starting from two weeks before Torpedo AChR (TAChR) immunization and continued till the end of experiments (Fig. 1A). The prophylactic effect of IRT5 probiotics administration against EAMG progression was analyzed by measuring weight change and clinical score compared with PBS treated group during the treatment periods (Fig. 1B). Oral administration of IRT5 probiotics significantly suppressed EAMG development by lowing clinical score compared with PBS control group at all the time points (Fig. 1B and Table 1). Compared with PBS treatment group, IRT5 probiotics treatment significantly reduced disease symptoms such as weak grip strength, hunched posture and tremor. As weight loss is one of the typical symptoms of EAMG, weight change between the treatments groups was compared (Table 1). Rats administered with IRT5 probiotics showed an increase in body weight compared with PBS treated group. Mean clinical score of IRT5 probiotics treated group was also lower than that of PBS treated group (Fig. 1B and Table 1). Since presence of complement at the NMJ and a loss of AChR content are the major pathogenic factors for initiation and progression of the EAMG [20], [21], we compared the levels of the complement and acetylcholine receptors at the NMJ. Muscle sections from PBS or IRT5 probiotics treated rats after 6 weeks of EAMG induction were prepared and incubated with fluorescent labeled anti-C5b-9 antibody or α-bungarotoxin to detect complement deposition and AChR level, respectively. Naïve healthy rats used as a control for AChR content did not show any presence of complements (lower panel in Fig. 1C). In accordance with clinical score, PBS treated control groups with higher clinical score (Fig. 1B) showed a clear presence of the complement deposits at the NMJ (green band in upper left panel in Fig. 1C), while IRT5 probiotics treated groups rarely show the presence of complement (middle left panel in Fig. 1C). In addition, IRT5 probiotics treated groups showed a clear staining of AChRs while PBS-treated sick rats showed a very weak signal due to the loss of AChR at the NMJ (middle panel in Fig. 1C). These results indicated that oral administration of IRT5 probiotics prevented the EAMG progression accompanied by reducing of complement-mediated loss of AChR at the NMJ.

**Table 1:** Effect of IRT5 probiotics treatment on EAMG.

	Clinical score in 6 weeks						Lymphocytes proliferation (cpm)
Treatment	0	1	2	>3	Mean Clinical Score	βWeight 0–6 wk(g)	
PBS	7/44	14/44	11/44	13/44	1.8	7±12.2	14065±2927
Pro	24/45	8/45	6/45	7/45	0.93	34.8±5.2	702±254

### Suppression of AChR-reactive Lymphocyte Response by IRT5 Probiotics

**Figure 2:**
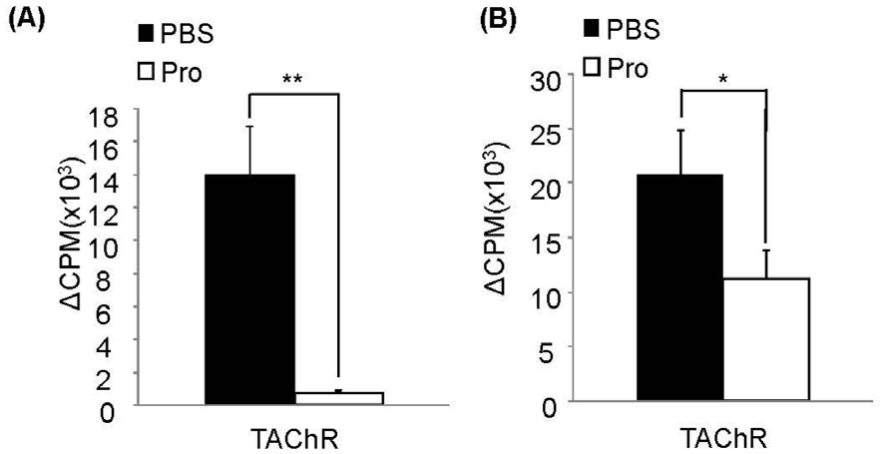
IRT5 probiotics treatment suppresses AChR-reactive lymphocyte proliferation. (A) Mixed lymphocytes and (B) CD4^+^ T cells of draining lymph nodes from each treatment group were cultured for 3 days either alone or presence of TAChR. Proliferation of mixed lymphocytes and CD4^+^ T cells were determined by assessing [^3^H]-thymidine incorporation during the last 18 hr of 72 hr culture period to evaluate proliferated status of cells. Results are expressed as CPM. *p<0.05, **p<0.01.

AChR-reactive T cells and B cells are responsible for progression and pathogenesis of MG and EAMG by producing inflammatory mediators. To investigate whether protective effect of IRT5 probiotics against EAMG progression is associated with alteration of AChR-reactive lymphocyte responsiveness, lymphocyte proliferation assay was performed. Draining lymph nodes (dLNs) were isolated from each treatment group six weeks after EAMG induction. Mixed lymphocytes and CD4^+^ T cells from dLNs were cultured for 3 days in the presence or absence of TAChR and their proliferation was measured by performing [^3^H] thymidine incorporation assay. To evaluate AChR-reactive CD4^+^ T cells proliferation, CD4^+^ T cells isolated from dLNs of each treatment group were co-cultured with normal rat splenocytes treated with mitomycin C either in the presence or absence of TAChR. Mixed lymphocytes (Fig. 2A) or CD4^+^ T cells (Fig. 2B) isolated from rats treated with IRT5 probiotics exhibited significantly lower proliferation compared with PBS treated groups. These results indicated that protective effect of IRT5 probiotics in EAMG progression is associated with AChR-reactive lymphocyte hypo-responsiveness.

### Suppression of Pro-inflammatory Cytokines by IRT5 Probiotics

**Figure 3:**
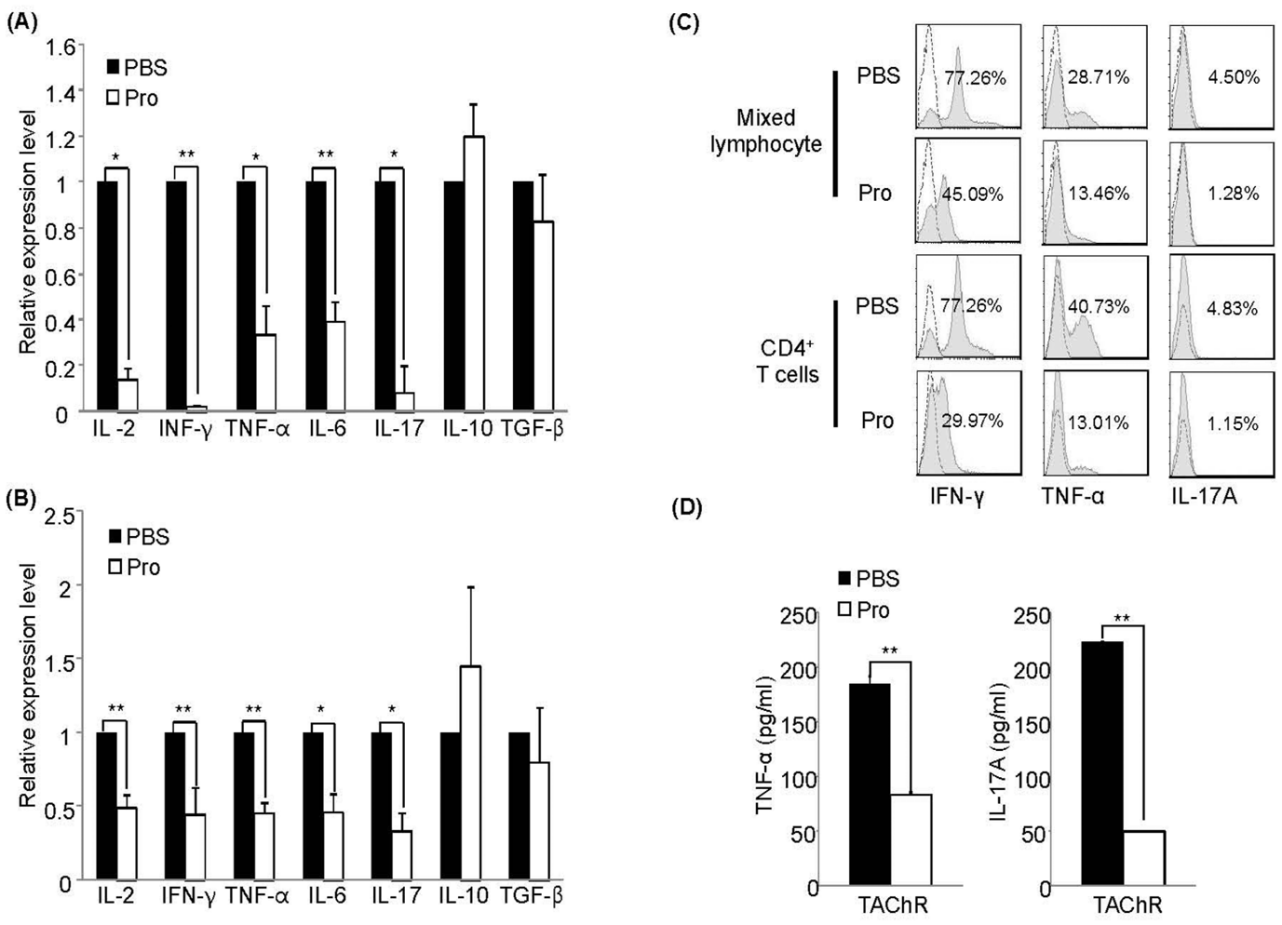
Treatment with IRT5 probiotics down-regulates expression of pro-inflammatory cytokines in draining LN. (A) Mixed lymphocytes and (B) CD4^+^ T cells isolated from the draining lymph nodes (dLN) of each treatment group were cultured for 40 hr in the presence of TAChR, and then total mRNA was isolated. The expression level of cytokines of control PBS group was set at 100% and the relative value of the IRT5 probiotics treated group was shown. (C) IFN-γ, TNF-α and IL-17A producing mixed lymphocytes and CD4^+^ T cells were analyzed by flow cytometry. (D) Protein levels of TNF-α and IL-17A from mixed lymphocytes were measured by ELISA. Data are representative of three independent experiments. *p<0.05, **p<0.01.

To further examine the effect of IRT5 probiotics on the levels of disease-associated cytokines, we measured the mRNA or protein levels in mixed lymphocytes and CD4^+^ T cells. Accumulating evidences suggested a pivotal role of pro-inflammatory cytokines such as IFN-γ and TNF-α in the development and the pathogenesis of EAMG [22], [23], [24], [25], [26], [27]. To examine cytokine expressions level, mixed lymphocytes and CD4^+^ T cells from dLNs of each treatment group were prepared 6 weeks after EAMG induction and stimulated for 2 days in the presence of TAChR. The relative mRNA expression level of pro- or anti-inflammatory cytokines were measured by quantitative real-time PCR normalized with ribosomal protein L32 gene. Compared with control PBS treated group, mixed lymphocytes and CD4^+^ T cells isolated from IRT5 probiotics treated rats showed much lower expression levels of IL-2 and pro-inflammatory cytokines such as, IFN-γ, TNF-α, IL-6 and IL-17 both in mixed lymphocytes (Fig. 3A) and CD4^+^ T cells (Fig. 3B). However, no significant alteration was observed in the expression levels of regulatory cytokines such as IL-10 and TGF-β. In accordance with mRNA data, IRT5 probiotics treatment significantly reduced the protein levels of IFN-γ, TNF-α and IL-17A in mixed lymphocytes and CD4^+^ T cells (Fig. 3C and 3D). IRT5 probiotics treatment also reduced the expression levels of pro-inflammatory cytokine expression in splenic lymphocytes (Fig. S1).

### Down-regulation of Anti-AChR Antibody Levels by IRT5 Probiotics

**Figure 4:**
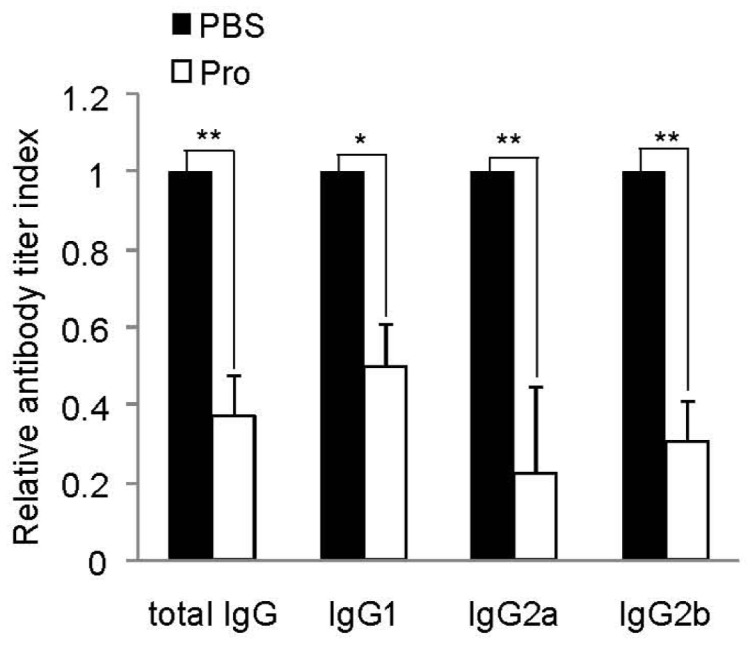
Treatment with IRT5 probiotics down-regulates AChR specific IgG isotypes. Sera were collected from EAMG rats treated with either IRT5 probiotics or control PBS for 6 weeks after immunization. Each AChR specific IgG isotype in the PBS-treated group was assigned a value of 1 and the ODs of IRT5 probiotics-treated group were calculated accordingly. Anti-AChR isotypes were determined by ELISA as described in the Material and Method. Data are representative of three independent experiments.*p<0.05, **p<0.01.

Anti-AChR specific antibodies play an important role in the pathogenesis of MG and EAMG through the recruitment of complement at the NMJ and functional blockade of acetylcholine binding to AChR [21], [28]. Passive transfer of AChR antibodies into experimental animals causes myasthenic symptoms [29]. Since AChR-reactive antibody production is affected by the type of activated CD4^+^ T cells, we tested whether IRT5 probiotics treatment affects AChR-reactive humoral immune responses. The anti-AChR IgG isotype levels were measured 6 weeks after the induction of EAMG by using human acetylcholine receptor fragment Hα1-205 as the plate coating antigen [30]. IRT5 probiotics-treated rats exhibited significantly lower levels of AChR-reactive total IgG, IgG1, IgG2a and IgG2b compared with PBS treated control rats (Fig. 4). These results suggested that oral administration of IRT5 probiotics lowered AChR-specific IgG and IgG isotypes levels, which mediates protective effect in EAMG progression.

### IRT5 Probiotics Generates Tolerogenic DCs in Mesenteric and Draining Lymph Nodes

**Figure 5:**
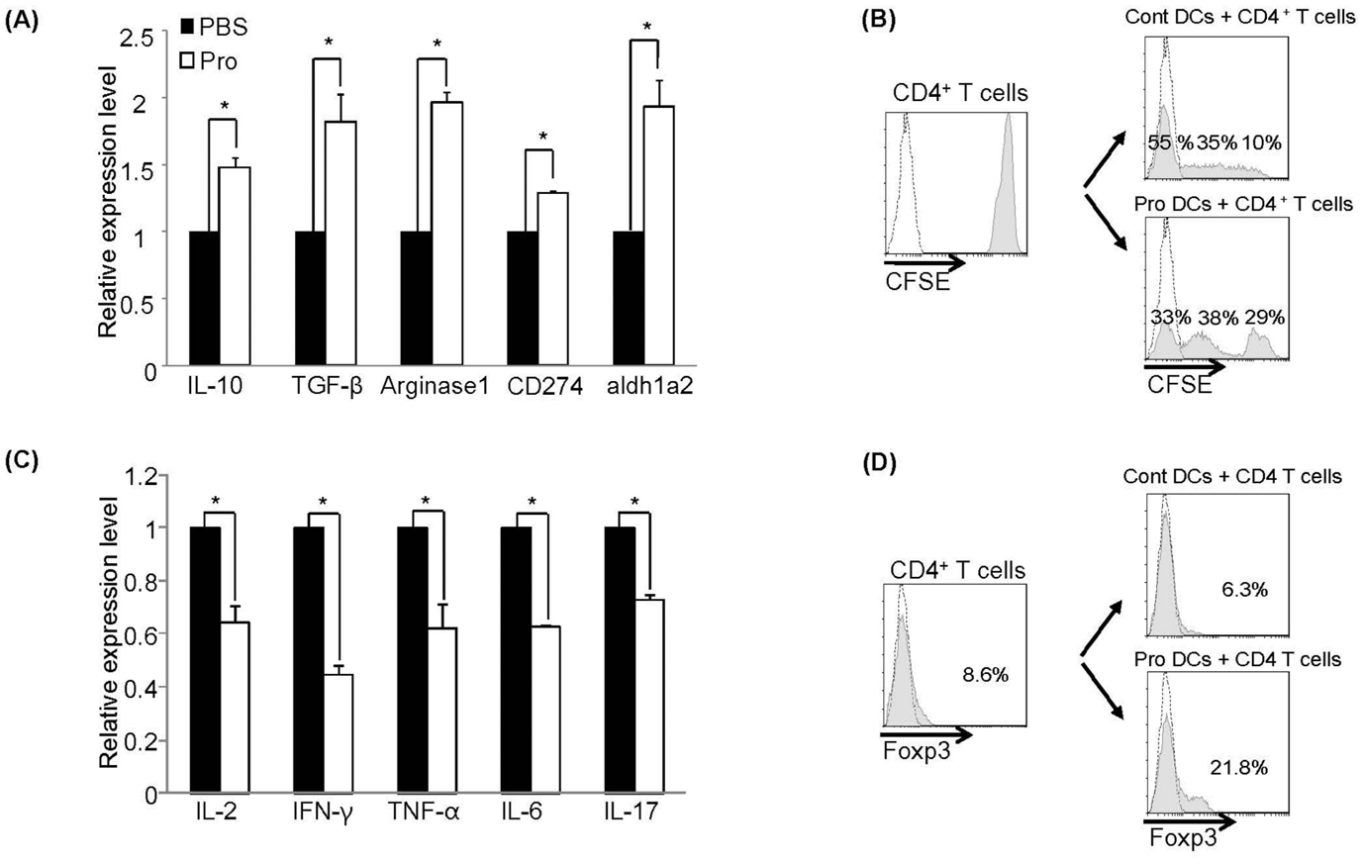
IRT5 probiotics administration induces regulatory DCs. (A) MLN DCs isolated from IRT5 probiotics or PBS treated groups were stimulated with PMA/Ionomycin and then the expression levels of marker molecules for rDC were measured by RT-PCR. (B) CFSE-labeled CD4^+^ T cells isolated from EAMG rats were stimulated by DCs isolated from each treatment group in the presence of TAChR and then proliferation (B) and production of cytokine levels (C) by CD4^+^ T cells were analyzed by flow cytometry and RT-PCR respectively. Proliferation of CD4^+^ T cells was monitored for 7 days co-culture periods. Cytokine expression in CD4^+^ T cells of PBS control mice was set at 100%. (D) CD4^+^ T cells isolated from EAMG rats were stimulated with MLN DCs from each treatment group in the presence of TAChR for 7 days. After co-culture, alteration of CD4^+^Foxp3^+^ population was measured by flow cytometry. The data are representative of three independent experiments. *p<0.05.

Mesenteric lymph nodes (MLN) is the site where the probiotics could exert their actions by affecting the mucosal antigen presenting cells such as dendritic cells (DCs) or macrophages. To further investigate underlying action mechanism of IRT5 probiotics on the suppression of EAMG progression, we analyzed the expression level of marker molecules for regulatory DCs such as IL-10, TGF-β, arginase1, CD274 (PDL-1), and aldh1a2. DCs were isolated using OX-62 labeled MACS bead in the MLN from IRT5 probiotics- or PBS-treated rats. Purified DCs were stimulated with PMA/ionomycin for polyclonal stimulation, total RNAs were extracted and the relative expression levels were analyzed by quantitative real-time PCR. Interestingly, compared with PBS treated group, IRT5 probiotics treated group showed much higher expression in the levels of all the tested molecules (Fig. 5A). To further investigate whether DCs from IRT5 probiotics treated rats have tolerogenic property, we performed DC-dependent T cell proliferation assay. CD4^+^ T cells from EAMG rats were pre-labeled with CFSE and co-cultured with DCs isolated from the MLN of each treatment group in the presence of TAChR. Seven days after the co-culture proliferation of DC-dependent AChR-specific T cell proliferation was monitored by flow cytometry. Proliferation of AChR-reactive CD4^+^ T cells was significantly lower in the co-cultured with DCs isolated from IRT5 probiotics-treated group compared with those of PBS-treated group (Fig. 5B). To further characterize the AChR-reactive CD4^+^ T cells isolated from the co-cultured with DCs, the expression profile of cytokine production was analyzed. The levels of IL-2 and pro-inflammatory cytokines such as IFN-γ, TNF-α, IL-6 and IL-17 were down-regulated in CD4^+^ T cells co-cultured with IRT5 probiotics treated DCs compared with PBS treated DCs (Fig. 5C).

**Figure 6:**
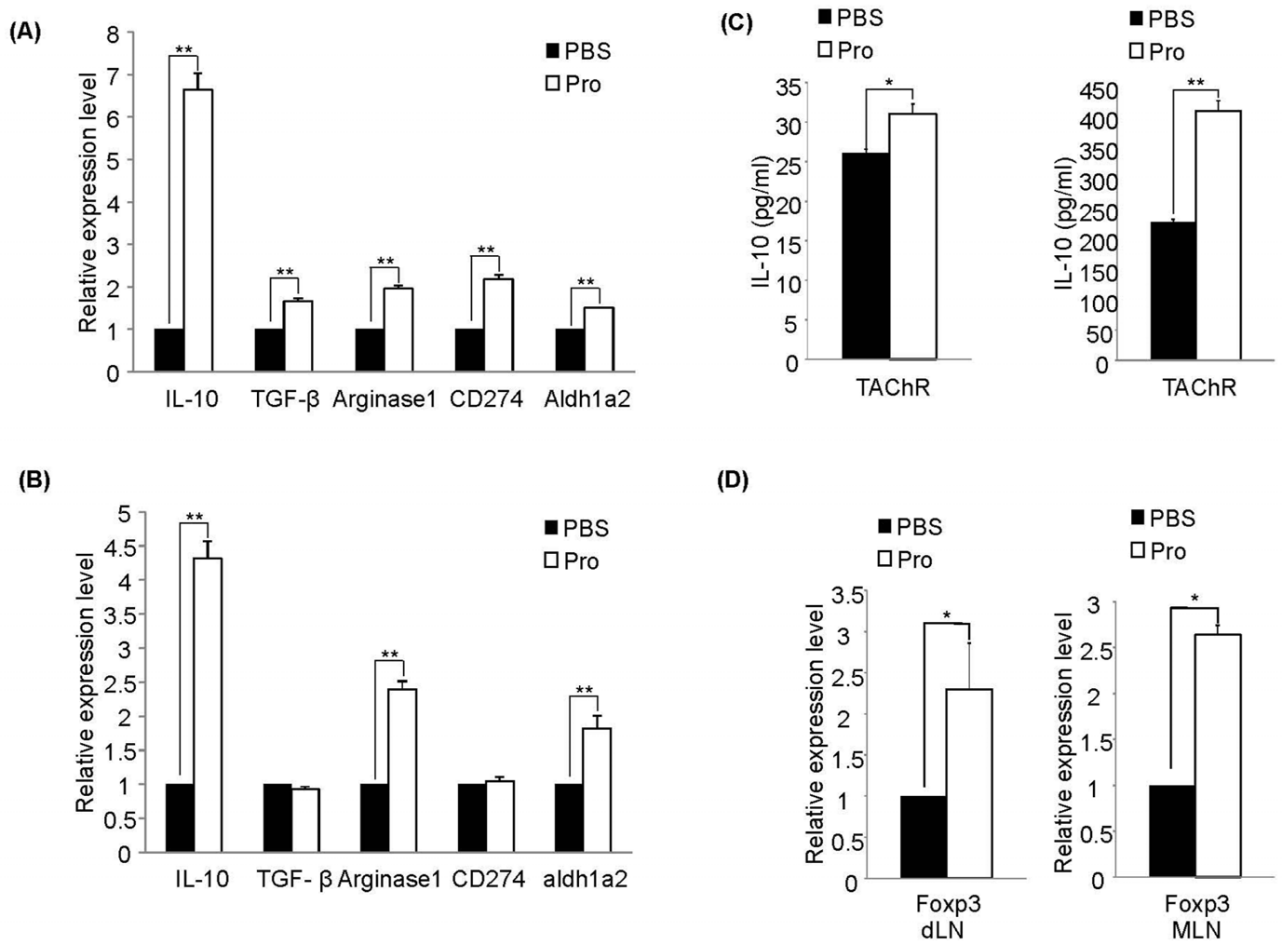
IRT5 probiotics administration induces regulatory DCs in the peripheral sites. (A) dLN DCs and (B) splenic DCs isolated from IRT5 probiotics or PBS treated group were stimulated with TAChR and then the expression levels of marker molecules for rDC or IL-10 protein expression (C) were measured by RT-PCR and ELISA, respectively. (D) Lymphocytes isolated from IRT5 probiotics or PBS treated groups were stimulated with TAChR and the relative expression levels of Foxp3 was measured by RT-PCR. The data are representative of three independent experiments. *p<0.05, **p<0.01.

It has been known that regulatory DCs (rDCs) have a potency to convert CD4^+^ T cells into Foxp3^+^ regulatory T cells (Tregs) [31]. We also analyzed whether down-regulation in the levels of pro-inflammatory cytokines by CD4^+^ T cells co-cultured from DCs isolated from IRT5 probiotics-fed rats is related with generation of rDC-mediated Tregs. CD4^+^ T cells were co-cultured with DCs from IRT5 probiotics- or PBS treated group for 7 days and then Foxp3^+^ population were analyzed by flow cytometry. Interestingly, the populations of CD4^+^Foxp3^+^ regulatory T cells were significantly increased in CD4^+^ T cells (21.8%) when cultured with DCs isolated from IRT5 probiotics-treated rats compared with control DCs (6.3%) (Fig. 5D). The value of mean fluorescence intensity (MFI) was 181 and 141 for IRT5 probiotics or PBS-treated group, respectively. We also investigated whether administration of IRT5 probiotics also generates regulatory DCs in the draining lymph nodes (dLN) and spleen. DCs isolated from dLN and spleen were stimulated with TAChR, total RNAs were extracted and the relative expression levels of rDC-associated molecules were analyzed by quantitative RT-PCR. dLN and splenic DCs from IRT5 probiotics treated group showed higher expression levels of IL-10, Arginase1 and aldh1a2 (Fig. 6A and B). Significant up-regulation of IL-10 protein level was observed in DCs from IRT5 treated groups compared to PBS treated groups (Fig. 6C). We tested whether regulatory DCs induced by IRT5 probiotics treatment also affect the generation of regulatory T cells. IRT5 probiotics treatment did not increase the CD4^+^Foxp3^+^ Treg cells in the *ex vivo* isolated cells (Fig. S3). However, upon AChR stimulation, significant up-regulation of CD4^+^Foxp3^+^ Treg was observed in both dLN and MLN (Fig. 6D). These results indicated that prophylactic effect of IRT5 probiotics is associated with the generation of rDC-mediated Foxp3^+^ regulatory T cells.

## Discussion

In this study, we have tested and elucidated the molecular mechanism of IRT5 probiotics-mediated suppression of experimental myasthenia gravis. Oral administration of IRT5 probiotics ameliorated the progression of EAMG by reducing AChR-reactive lymphocyte proliferation, AChR antibody levels and pro-inflammatory cytokine levels. In addition, administration of IRT5 probiotics generated regulatory DCs in MLN, dLN and spleen that have a potency to increase CD4^+^Foxp3^+^ Tregs.

Recently, probiotics become the center of public interest based on the finding that it can provide beneficial effects on host’s health by various mechanisms. Probiotics intakes improve microflora function, decrease serum cholesterol level and prevent cancer activity [32], [33], [34], [35]. Probiotics modulate mucosal immune system by removing pathogens from the intestine or affecting intestinal epithelial cells and immune cells. For example, probiotics prevent colonization of pathogens in the mucosal territory [36]. A number of accumulating studies showed that administration of probiotics are effective for inflammatory bowel disease (IBD). Treatment with probiotics exhibited the beneficial effects on the remission of ulcerative colitis (UC) and prevented the relapse of pouchitis in UC patients. Consumption of probiotics increased the regulatory T cells and decreased TNF-α and IL-12 producing macrophages and DCs in the peripheral blood of IBD patients [37], [38], [39]. Ingestion of probiotics could modulate autoimmune diseases such as rheumatoid arthritis [3], [40], [41], type I diabetes [41], and experimental autoimmune encephalomyelitis (EAE) [42], which are mainly mediated by pro-inflammatory cytokines. In addition, probiotics ameliorated atopic dermatitis in which Th2 type cytokines have crucial pathogenic roles [5]. However, the therapeutic or prophylactic effects of probiotics on various diseases depend on the strains, administered routes, doses and the disease states when they treated [43]. In addition, one of the most challenging questions in probiotics induced immune modulation is how to identify or select a certain strain with potent immune modulating properties. Recently, we have developed a novel screening system to select probiotics or their mixture to enhance the generation of CD4^+^ Foxp3^+^ Tregs cells [13]. Oral administration of the IRT5 probiotics, a mixture of five probiotics, exerted therapeutic efficacy in experimental models of colitis (IBD), atopic dermatitis (AD) and rheumatoid arthritis (RA) [13].

In this study, we tested the immunomodulatory effect of IRT5 probiotics on EAMG development and elucidated underlying mechanism of action involved in EAMG regression. Oral administration of IRT5 probiotics significantly reduced the EAMG symptoms (Fig. 1B and Table 1) by inhibiting the infiltration of complement component and loss of AChR contents at NMJ (Fig. 1C). Treatment with IRT5 probiotics further suppressed AChR reactive immune responses and down-regulated the levels of pro-inflammatory cytokines (IFN-γ, TNF-α, IL-6 and IL-17). In addition, oral administration of IRT5 probiotics reduced the anti-AChR IgG isotype levels such as IgG1, IgG2a and IgG2b (Fig. 4). IgG isotypes are associated with T helper cells and complement involvement. Antigenic modulation is an essential effector mechanism of autoantibodies in MG that down-regulate the AChR [44]. Among the different IgG isotypes, the concentration of IgG1 is related to Th2-type inflammatory immune responses, whereas IgG2a, IgG2b are known to be mediators of Th1-type inflammation and associated with the pathogenesis of myasthenia gravis and other autoimmune diseases [30], [45]. IgG2 subclasses are involved in complement-fixing activity which has pathogenic roles in neuromuscular junction of myasthenia gravis in mice [18], [22], while IgG1 subclass was highly observed with beneficial effect on preventing generation of autoimmune diseases [46], [47]. However, the role of IgG isotype in MG or EAMG is still controversial, since the rat IgG1 also activates complement [48]. In humans it is unclear how the immune response can be modulated to induce protective IgG4 antibodies [48]. Interestingly, IRT5 probiotics treatment significantly lowered all the IgG isotype levels without inducing IgG isotype switching from Th1 (IgG2a and IgG2b) to Th2 (IgG1) phenotype (Fig. 4).

It is well reported that pro-inflammatory cytokines such as IFN-γ and TNF-α mainly produced by Th1 cells play an essential role on the development and induction of EAMG [25], [49]. Recently, several groups have reported that Th17 cells and their cytokines are also involved the pathogenesis of EAMG [50], [51]. Interestingly, treatment with IRT5 probiotics significantly suppressed IL-17 expression in total lymphocytes (Fig. 3A and 3C) as well as in CD4^+^ T cells (Fig. 3B and Fig. 3D) without enhancing IL-10 or TGF-β levels. These results imply that oral administration of IRT5 probiotics mainly inhibited inflammatory T helper cell functions rather than inducing phenotypic switching from Th1 cytokines to Th2/Th3 cytokines.

How does IRT5 probiotics induce AChR-reactive lymphocyte tolerance? Probiotics which enter the gastrointestinal tract through administration by oral route can modulate or affect the immune cells beneath the intestinal epithelial cells. Probiotics can also cross the intestinal epithelial cells directly via DCs that could capture the probiotics by using their dendrites [52]. Probiotics which transverse the gut lumen stimulate the antigen presenting cells especially macrophages or DCs to produce cytokines such as IL-12, IL-10 and TGF-β [53], [54]. Administration of probiotics has not only local (gut and colon) but also systemic effect on various disease conditions [55], [56]. In our previous studies in mouse system, we have demonstrated that protective effect of IRT5 probiotics in IBD and AD is associated with generation of regulatory DCs (rDCs), which in turn promoted the generation of CD4^+^Foxp3^+^ T cells. However, no information is available whether administration of certain probiotics can generate DCs with tolerogenic property in rat. In this study, we demonstrated that oral administration of IRT5 probiotics can generate rDCs in mesenteric lymph node (MLN), draining lymph node (dLN) and spleen. However, it is still unclear how precisely probiotics induced rDC generation, in addition to their origin. They might be generated in the MLN and then a proportion of them migrated to the dLN and spleen. However, we believe that this type of detailed investigation lies beyond the scope of our current study. Compared with PBS-treated group, DCs isolated from IRT5 probiotics treated rat have rDC properties confirmed by high expression of rDC marker molecules (IL-10, TGF-β, arginase1, PDL-1 and aldh1a2) [57] (Fig. 5A, 6A and 6B) and their capacity to generate CD4^+^Foxp3^+^ Tregs (Fig. 5D) to suppress AChR-reactive T cells proliferation (Fig. 5B). These results collectively suggest that the observed suppression of AChR-reactivity in mixed lymphocyte as well as in CD4^+^ T cells (Fig. 2) and down-regulation of proinflammatory cytokines (Fig. 3) by IRT5 probiotics are mediated by the generation of regulatory DCs.

Based on the prophylactic effect of IRT5 on the progression of EAMG, we further tested whether administration of IRT5 could reverse the ongoing stage of EMAG. However, when we orally administered IRT5 probiotics during acute stage of EAMG development, we did not observe therapeutic effect against EAMG progression (Fig. S2 and Supplementary Table 1). There are several possibilities for this failure. First, although IRT5 administration could induce regulatory DCs in MLN, dLN and spleen, their potency to convert effector CD4^+^ T cells into regulatory T cells is still limited. Indeed, we could not observe upregulated Treg in the absence of antigen specific stimulation condition although IRT5 administration still exert prophylactic effect on EAMG progression. Second, administration of probiotics can modulate more easily the naïve state immune cells than the pre-activated or lineage committed inflammatory cells. Administration of IRT5 probiotics induced similar phenotypes of rDCs in both the healthy and EAMG rat (Fig. S4). However, already differentiated or activated auto-reactive T or B cells will not easily be altered by external immune modulators. Indeed, administration of IRT5 probiotics during ongoing EAMG significantly reduced the levels of anti-AChR total IgG and IgG isotypes (Fig. S2D). Previous studies also showed that these treatments are ineffective to ameliorate the clinical symptoms in the EAMG model, even if the autoantibody levels are reduced [58]. In order to overcome this problem, we need to consider co-administration of probiotics with immunotherapeutic agent like oral tolerogen. We have previously reported this possibility that co-administration of probiotics with oral tolerogen has more potent therapeutic effect on the experimental rheumatoid arthritis compared probiotics or oral tolerogen alone [59], [60]. In the future, we will test whether oral administration of IRT5 probiotics with recombinant AChR [30], [45] as a tolerogen could suppress ongoing EAMG.

In conclusion, we have demonstrated that pretreatment of IRT5 probiotics before disease onset significantly suppressed development of EAMG by inhibiting AChR-reactive lymphocyte responses though generation of regulatory DCs. Although probiotics alone may have limited capacity to modulate ongoing EAMG progression, co-administration of other therapeutic modality may lead to suppress AChR-reactive lymphocytes in disease progression.

## Materials and Methods

### Animals

Female Lewis rats 6–8 weeks of age (160 g–180 g) were obtained from Charles River Japan, Inc. (Shiga, Japan) and were housed in specific pathogen-free barrier facilities. Animal experiments performed were approved by the Animal Care and Use Committees (ACUC) of the Gwangju Institute of Science and Technology (authorization number GIST-2008-11 from ACUC).

### Induction and Clinical Evaluation of EAMG

EAMG was generated in female Lewis rats by the immunization of Torpedo acetylcholine receptors (TAChRs) obtained from electric organs of Torpedo California by affinity chromatography as previously described [47]. Briefly, rats were immunized once in base tail by injection of purified TAChR (20–40 βg/rat) with emulsified in complete Freund’s Adjuvant (CFA) (BD Biosciences, NJ). Weight changes of EAMG were monitored on three times per week and clinical symptoms of EAMG were graded 0–4 based on the observation before and after exercise as following; Grade 0, rat with normal posture, muscle strength no symptoms of EAMG; grade 1, normal at rest, slightly decreased activity and grip strength with hunched posture after exercise; Grade 2, hunched posture at rest, weakness, loss of body weight and tremor; grade 3, severe weakness, dramatically decreased body weight and moribund; grade 4, dead. Clinical score were assessed by double blind evaluation for 6 weeks following the TAChR immunization. All experiments groups consisted of 8–9 rats.

### Preparation of Probiotics Mixture and Oral Administration of Probiotics Mixture

The IRT5 probiotics powder contains 1×10^11^ cfu/g of each strain consisting of *Lactobacillus casei*, *Lactobacillus acidophilus*, *Lactobacillus reuteni*, *Bifidobacterium bifidum*, and *Streptococcus thermophilus*. All the probiotics strains were kindly provided by Korea Yakult Co. (Giheung, Korea). To test the preventive effects IRT5 probiotics rats were orally administrated five times per weeks starting from two weeks before TAChR immunization and continued till the end of experiments (6–8 weeks after immunization). To test the therapeutic effect of IRT5 probiotics, treatment was initiated one week after TAChR immunization and continued until the end of experiment. The amounts of probiotics were 2×10^9^ cfu/each strain in 1 ml PBS per rat.

### Cell Culture and Cell Stimulation

CD4^+^ T cells were purified from draining lymph nodes by CD4 specific immunomagnetic beads according to the manufacturer’s instructions (Miltenyi biotec, Auburn, CA). Isolated CD4^+^ T cells were cultured in T cell medium containing RPMI (Invitrogen, Grand Island, NY) supplemented with 10% fetal bovine serum (Hyclone Laboratories, Logan, USA), 3 mM L-glutamine (Sigma-Aldrich; St. Louis, MO), 10 mM HEPEs (Sigma-Aldrich; St. Louis, MO), 100 U/ml penicillin (Sigma-Aldrich; St. Louis, MO) and 100 U/ml streptomycin (Sigma-Aldrich; St. Louis, MO) and 0.05 mM 2-β-mercaptoethanol (Sigma-Aldrich; St. Louis, MO). For cytokine analysis, mixed lymphocytes and CD4^+^ T cells were stimulated with TAChR.

### Anti-AChR Antibody and AChR-specific IgG Isotyping

Sera were obtained from EAMG rats after 24 hours of last feeding by retro-orbital bleeding. Anti-rat AChR antibody levels were measured by ELISA as follows: 96-well microplates were coated with Hα1-205, a recombinant fragment corresponding to extracellular human AChR α subunit (100 βL; 20 βg/mL) [47] and reacted with 100 βL of the tested serum samples at proper dilution (1/10000 for total IgG, 1∶500 for IgG1, 1∶1000 for IgG2a and IgG2b). Horseradish peroxidase conjugated mouse mAbs to Rat IgG isotypes (1/50,000 for total IgG, 1/60,000 for IgG1, 1/20,000 for IgG2a and IgG2b) were added for 2 hrs at room temperature, and bounded antibodies were detected by the activity of the peroxidase. Antibody levels were evaluated by measuring the OD at 405 nm.

### CD4^+^ T Cells, Mixed Lymphocytes Proliferation Assay

Draining lymph node cells (dLNCs) from IRT5 probiotics or PBS treated EAMG rats were collected and cultured in T cell medium either alone or in the presence of TAChR. CD4^+^ T cells from IRT5 probiotics or PBS treated EAMG draining lymph node were co-cultured with allogeneic rat splenocytes treated with mitomycin C either alone or in the presence of TAChR. Proliferation was assessed by measuring [^3^H] thymidine (0.5 βCi/well) incorporation during the last 18 h of 72 hrs culture period. Results are expressed as Δcpm after subtraction of background of unstimulated cultures from stimulated dLNCs.

### Determination of Cytokine Levels by Quantitative Real-time PCR and ELISA

In each tested groups, three rats of representative mean clinical score were sacrificed. dLNCs and CD4^+^ T cells isolated from these groups were culture in the presence of TAChR as a stimulant for 40 hrs and harvested. Total RNA was extracted by using TRIzol reagent (Molecular Research Center; Cincinnati, OH), according to the manufacturer’s protocol and was reverse transcribed to prepare cDNA using M-MLV reverse transcriptase (Promega, Madison, WI). Prepared cDNA was subjected to quantitative real time-PCR using Chromo-4 (Bio-Rad, Hercules, CA) with SyBr Premix Ex tag (Takara, Japan). Specific primer pairs are described at Table 2. The data was normalized by expression level of L-32 while calculating the expression levels of other genes. Results are described as a relative expression level for each gene in the experimental group compared with that of control group. For the analysis of protein expression level, mixed lymphocytes from dLN and splenocytes were stimulated with TAChR for 96 hrs. The level of cytokines in the culture supernatants was determined by ELISA with specific antibodies for TNF-α and IL-17A, according to the manufacturer’s instructions (eBioscience, (San Diego, CA)).

**Table 2:** Primer sequences used for qRT-PCR.

Genes	Sequences (5′ –3′)	
L32	F: AGTTTCTGGTCCACAATG	R: GTTGGGATTGGTGACTCT
IL-2	F: CCTGCAAGCATGTACAGC	R: CTTGTAATTATCGATCCCTCTCAG
IL-6	F: ATATACCACTTCACAAGTCGG	R: GGCAAATTTC-CTGGTTATATCC
IFN-β	F: GAGCCTCCTCTTGGATATCTG	R: GTTGTTCACCTCGAACTTGG
TNF-β	F: AACAAGGAGGAGAAGTTCCC	R: GTTGTCTTTGAGATCCATGCC
IL-17	F: CATCCATGTGCCTGATGC	R: TCTGGAGAAAGTTATTGGCCT
IL-10	F: AACAAGGAGGAGAAGTTCCC	R: TGTATCCAGAGGGTCTTCAG
TGF-β	F: TGGAAATCAATGGGATCAGTC	R: GTAGTTGGTATCCAGGGCTC
Arginase1	F: CAAGACAGGGCTACTTTCAG	R: TCGGTGGTTTAAGGTAGTCAG
PDL-1	F: GAAAGTCAACGCTCCATACC	R: GTCACTGTTTGTCCAGATCAC
Aldh1a2	F: GCCAATAACTCAGACTTCGG	R: GCCAAACTCACCCATTTCTC

### Flow Cytometric Analysis of Intracellular Molecules

The expression levels of intracellular IFN-γ, TNF-α and IL-17A protein in antigen specific AChR-stimulated dLNC and CD4^+^ T cells were analyzed by flow cytometry. In brief, mixed lymphocytes from dLNC and spleen were cultured for 4 days in the presence of TAChR. Mixed lymphocytes were re-stimulated with Phorbol 12-myristate 13-acetate (PMA) and ionomycin for 5 hr with 1 mg/ml Bredfeldin A and tracked by intracellular cytokine staining (ICS). Cells were harvested, washed with PBS, and stained with FITC labeled CD4 antibody for 20 min. Cells were washed with PBS and fixed in fixation/permeabilization buffer (eBioscience, (San Diego, CA)) for 30 min. Cells were washed with PBS and resuspended in permeabilization buffer (eBioscience, (San Diego, CA)). For intracellular detection of IFN-γ, TNF- α and IL-17A, anti-IFN-γ- PE (BD Biosciences, NJ), anti-TNF-α-PE (BD Biosciences, NJ) and anti-IL-17A-PE or isotype control Ab (eBioscience, (San Diego, CA)) were added and incubated for 30 min at 4°C. After incubation, cells were washed, resuspended in PBS and at least 10,000 cell events were evaluated using EPICS XL™ and EXPO32™ software (Beckman Coulter, CA, USA).

### Isolation of Dendritic Cells (DCs) and DC-induced T Cell Differentiation

Dendritic cells (DCs) were isolated from mesenteric lymph node (MLN), draining lymph node (dLN) and spleen by using anti-OX-62 magnetic beads (Miltenyi biotec, Auburn, CA) from IRT5 probiotics or PBS treated rat. Purified DCs were directly analyzed or stimulated with PMA/ionomycin or TAChR for determining cytokines and immunomodulatory molecules profile. For DC-mediated T cell differentiation, MLN DCs were co-cultured with CD4^+^ T cells isolated form EAMG rats in the presence of TAChR for 2 days. After co-culture, total RNA was extracted and cDNA were synthesized for determining cytokine profiles. Proliferative properties of AChR-reactive CD4^+^ T cells were evaluated by the CSFE labeling experiment. CD4^+^ T cells from EAMG rats were labeled with CSFE by following manufacturer’s instruction, and the labeled CD4^+^ T cells were co-culture with MLN DCs from IRT5 probiotics or PBS treated rat in the presence of TAChR. After 7 days co-culture, proliferation of CD4^+^ T cells were analyzed by flow cytometry. The population of CD4^+^Foxp3^+^ Treg cells generated in the co-culture condition was analyzed by flow cytometry.

### Immunohistochemistry Analysis of AChR and Complement Contents at the Neuromuscular Junction

Femurs and tibias muscle tissues from the non-immunized normal rats or IRT5 probiotics- or PBS-treated EAMG rats were extracted and embedded in Tissue-Tek OCT compound and sectioned in the transverse direction into 10 βm using a Jung Frigout 2800E Kryostat (Leica Camera, AGVertrieb, Deutschland). For each experimental group, 10–15 muscle sections were analyzed as previously described [61], [62]. Muscle sections were fixed in cold acetone for 10 min and washed them in PBS three times for 5 min. Sections were blocked with 2% PBSA (bovine serum albumin in PBS) for 90 min at room temperature, and then washed in PBS three times for 15 min. Sections were incubated for overnight at 4°C with tetramethylrhodamine-labeled α-BTX (1/500 in PBSA; Molecular Probes) or mAb 2A1 against rat C5b-9 (membrane attack complex, 1/100 in PBSA; Hycult biotech, PB UDEN, The Netherlands). Sections were then washed with PBS containing 0.05% Triton X-100 and were incubated with for 90 min at room temperature with secondary antibody; Alexa 488-conjugated goat anti-mouse (1/1000 in PBSA; Molecular Probes). Finally sections were washed in PBS three times for 15 min and then cover-slips were mounted with Dako mounting solution.

### Statistical Analysis

Data are the mean of SE of at least three independent experiments, unless differently specified in the text. The student′s *t*-test was used to determine the significance of the results. Differences were considered to be statistically significant when the P value was <0.05. Significance was only indicated when appropriate.

## Supporting Information

Figure S1 **Treatment with IRT5 probiotics down-regulates the expression levels of pro-inflammatory cytokines in the spleen.** (A) IFN-γ, TNF-α and IL-17A producing mixed lymphocytes and CD4+ T cells in spleen were analyzed by flow cytometry. (B) Protein levels of TNF-α and IL-17A from splenic mixed lymphocytes were measured by ELISA. Data are representative of three independent experiments. **p<0.01.
(TIF)

Figure S2 **No therapeutic effect of probiotics administration in the ongoing EAMG.** Oral administration of IRT5 probiotics or PBS as a control was initiated one week after TAChR immunization and continued until the end of experiment (8 weeks after induction of EAMG) (A). The therapeutic effect of IRT5 probiotics or PBS treatment was analyzed by monitoring clinical scores (B). Mean clinical score was evaluated based on the standard clinical scoring criteria. The points and bars represent means and standard deviations, respectively. Data are representative of two independent experiments. (C) Mixed lymphocytes in draining lymph nodes (dLN) isolated from each treatment group were cultured for 40 hr in the presence of TAChR, and then total mRNA was isolated. The expression level of cytokines in the control PBS group was set at 100% and the relative value of the IRT5 probiotics treated group was shown. (D) Sera were collected from EAMG rats treated with either IRT5 probiotics or control PBS for 6 weeks after immunization. Each AChR specific IgG isotype in the PBS-treated group was assigned a value of 1 and the ODs of IRT5 probiotics-treated group was calculated accordingly. Anti-AChR isotypes were determined by ELISA as described in the Material and Method. Data are representative of three independent experiments.*p<0.05, **p<0.01.
(TIF)

Figure S3 **No significant differences in the populations of** ***ex vivo*** **Treg cells.** Foxp3^+^ Treg population were analyzed from the *ex vivo* lymphocytes in the dLN and MLN. No significant difference was observed between the PBS and IRT5 probiotics treated groups. The data are representative of three independent experiments.
(TIF)

Figure S4 **Treatment of IRT5 probiotics generates regulatory DCs in the normal healthy and EAMG condition.** Relative expression levels of rDC marker molecules were compared between the MLN DCs of healthy (A and C) or EAMG (B and D) rats after treatment with PBS or IRT5 probiotics. IL-10 protein expression of MLN DCs isolated from healthy (E) and EAMG (F) were also measured by ELISA. Data are representative of three independent experiments.*p<0.05, **p<0.01.
(TIF)

Table S1 **Effect of IRT5 probiotics treatment on ongoing EAMG.**
(DOC)
