# Supplementary figures and images for: Prophylactic Effect of Probiotics on the Development of Experimental Autoimmune Myasthenia Gravis
Source: PLoS One. 2012 Dec 20;7(12):e52119. doi: 10.1371/journal.pone.0052119 (PMC3527378; doi:10.1371/journal.pone.0052119)

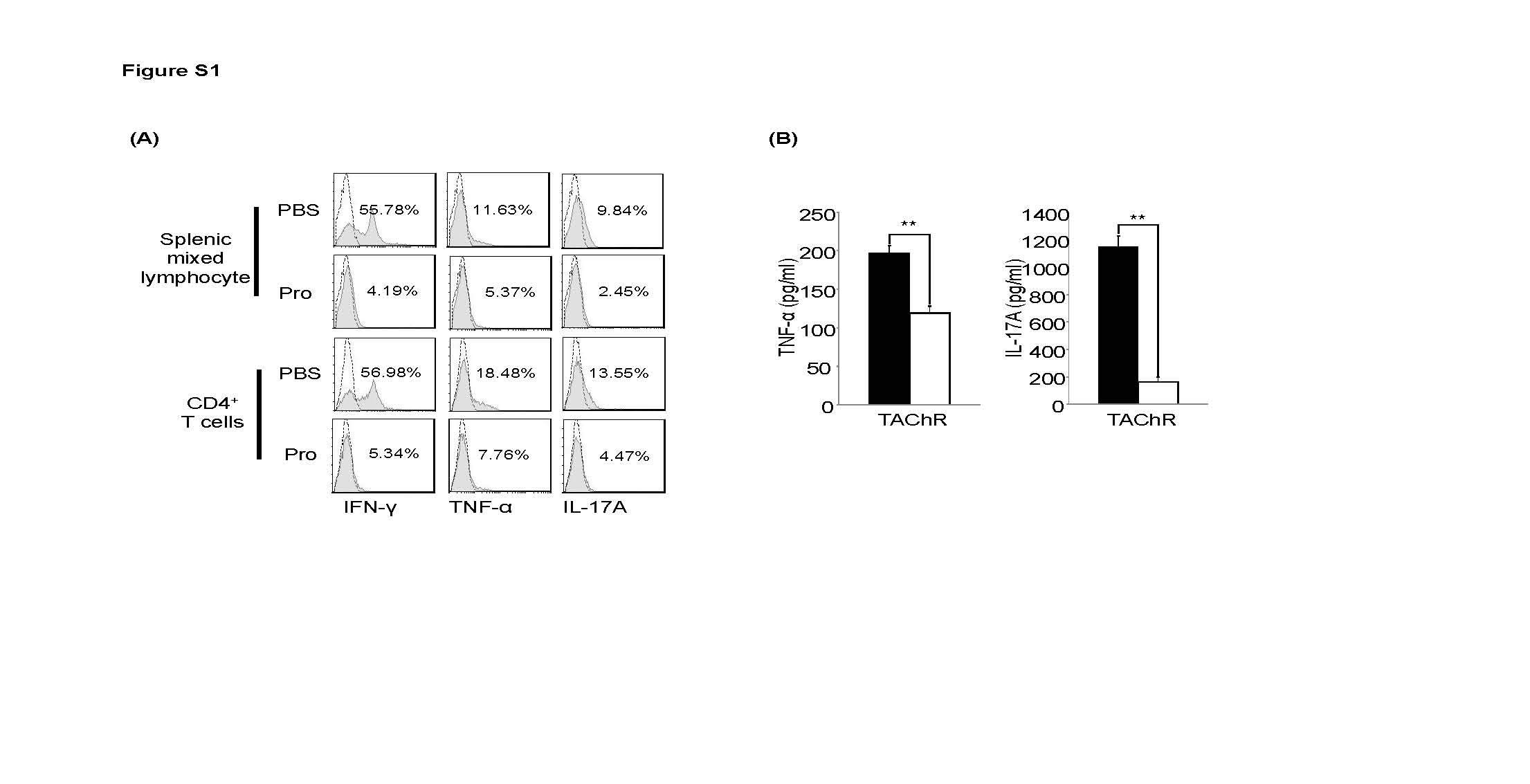

Supplement: Figure S1 — Treatment with IRT5 probiotics down-regulates the expression levels of pro-inflammatory cytokines in the spleen. (A) IFN-γ, TNF-α and IL-17A producing mixed lymphocytes and CD4+ T cells in spleen were analyzed by flow cytometry. (B) Protein levels of TNF-α and IL-17A from splenic mixed lymphocytes were measured by ELISA. Data are representative of three independent experiments. **p<0.01. (TIF) [file pone.0052119.s001.tif]

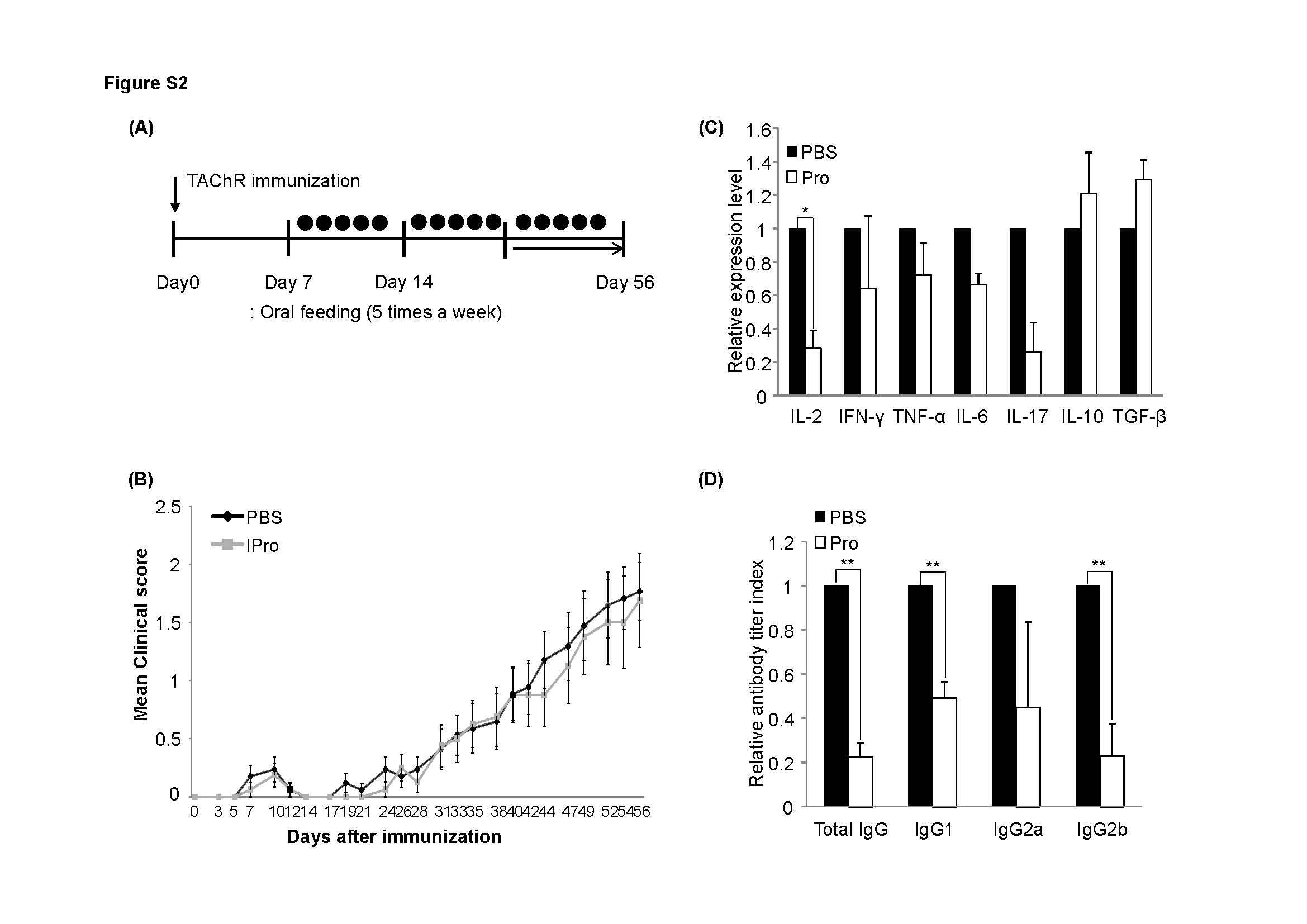

Supplement: Figure S2 — No therapeutic effect of probiotics administration in the ongoing EAMG. Oral administration of IRT5 probiotics or PBS as a control was initiated one week after TAChR immunization and continued until the end of experiment (8 weeks after induction of EAMG) (A). The therapeutic effect of IRT5 probiotics or PBS treatment was analyzed by monitoring clinical scores (B). Mean clinical score was evaluated based on the standard clinical scoring criteria. The points and bars represent means and standard deviations, respectively. Data are representative of two independent experiments. (C) Mixed lymphocytes in draining lymph nodes (dLN) isolated from each treatment group were cultured for 40 hr in the presence of TAChR, and then total mRNA was isolated. The expression level of cytokines in the control PBS group was set at 100% and the relative value of the IRT5 probiotics treated group was shown. (D) Sera were collected from EAMG rats treated with either IRT5 probiotics or control PBS for 6 weeks after immunization. Each AChR specific IgG isotype in the PBS-treated group was assigned a value of 1 and the ODs of IRT5 probiotics-treated group was calculated accordingly. Anti-AChR isotypes were determined by ELISA as described in the Material and Method. Data are representative of three independent experiments.*p<0.05, **p<0.01. (TIF) [file pone.0052119.s002.tif]

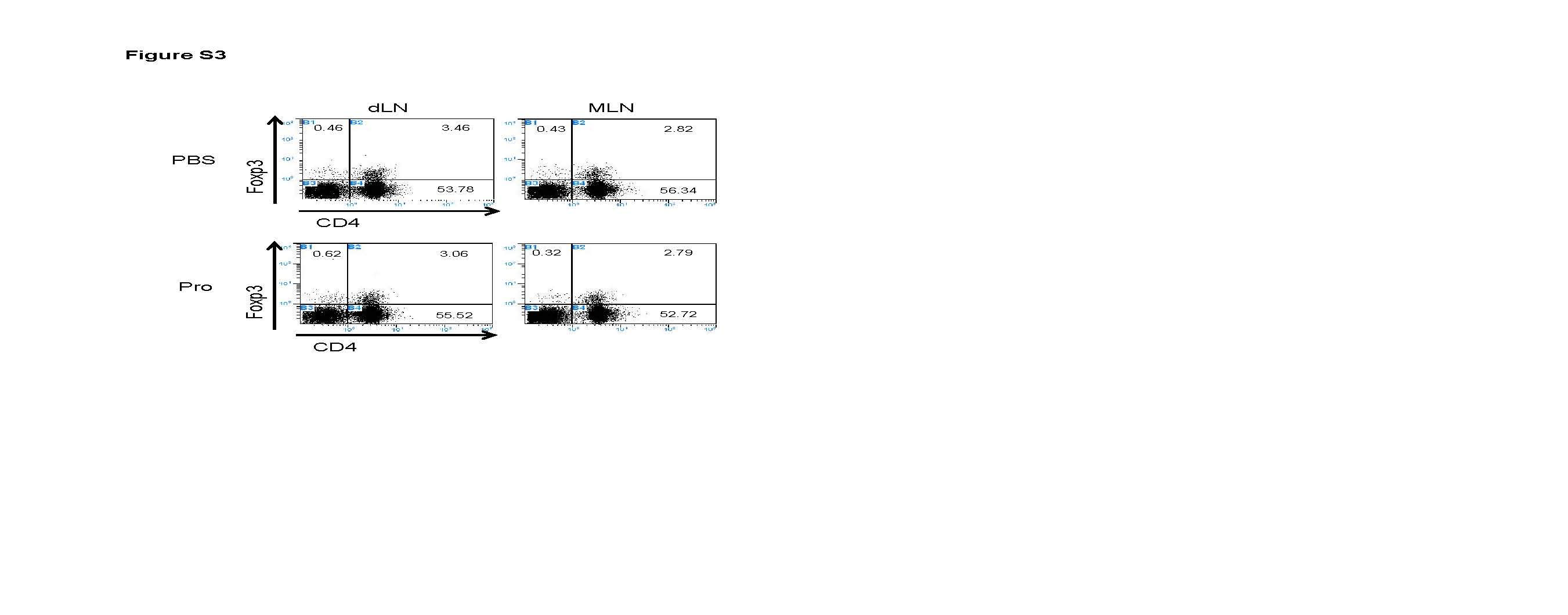

Supplement: Figure S3 — No significant differences in the populations of ex vivo Treg cells. Foxp3+ Treg population were analyzed from the ex vivo lymphocytes in the dLN and MLN. No significant difference was observed between the PBS and IRT5 probiotics treated groups. The data are representative of three independent experiments. (TIF) [file pone.0052119.s003.tif]

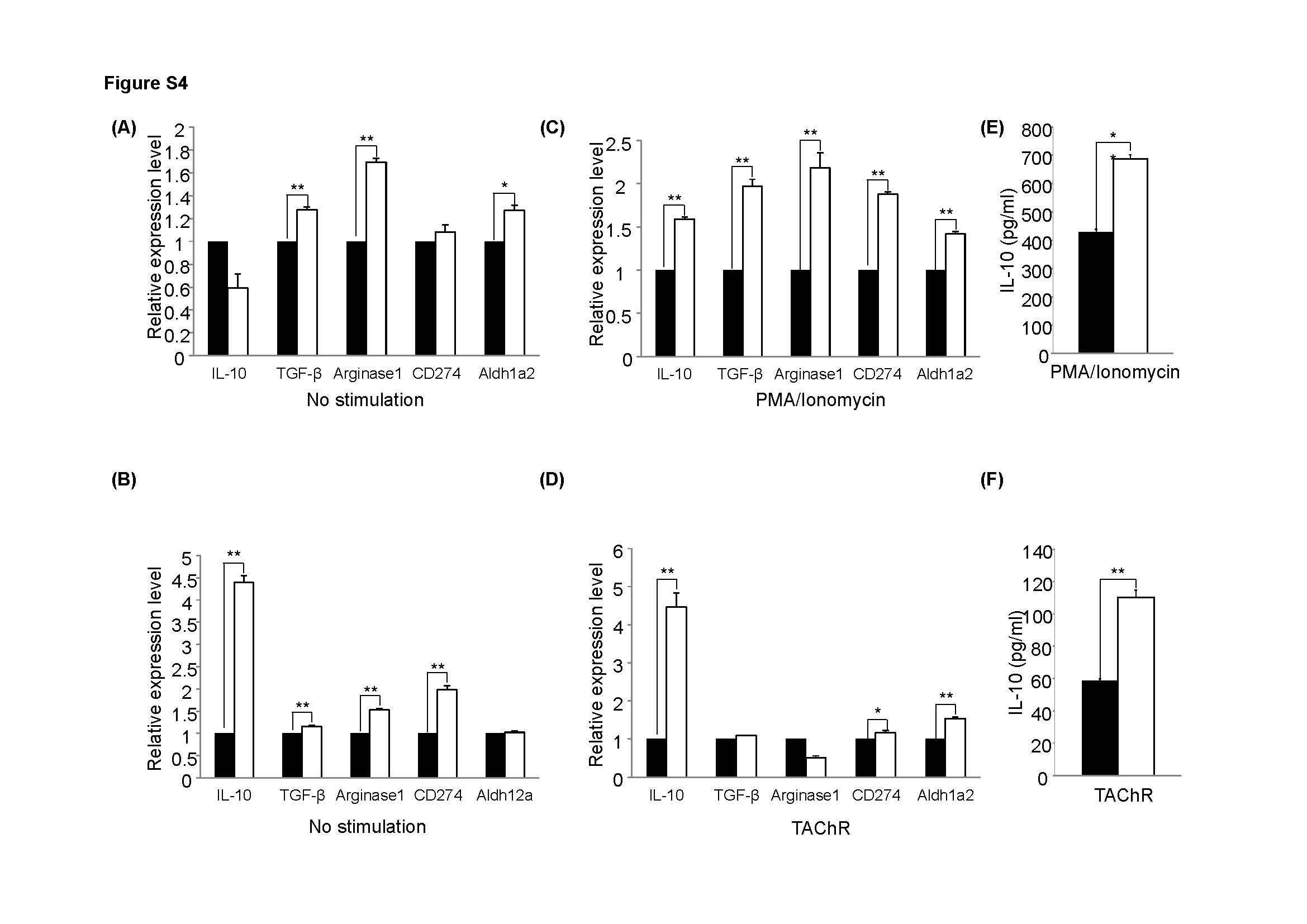

Supplement: Figure S4 — Treatment of IRT5 probiotics generates regulatory DCs in the normal healthy and EAMG condition. Relative expression levels of rDC marker molecules were compared between the MLN DCs of healthy (A and C) or EAMG (B and D) rats after treatment with PBS or IRT5 probiotics. IL-10 protein expression of MLN DCs isolated from healthy (E) and EAMG (F) were also measured by ELISA. Data are representative of three independent experiments.*p<0.05, **p<0.01. (TIF) [file pone.0052119.s004.tif]
