# Supplementary material for: Prophylactic Effect of Probiotics on the Development of Experimental Autoimmune Myasthenia Gravis
Source: PLoS One. 2012 Dec 20;7(12):e52119. doi: 10.1371/journal.pone.0052119 (PMC3527378; doi:10.1371/journal.pone.0052119)
Supplement: Table S1 — Effect of IRT5 probiotics treatment on ongoing EAMG. (DOC) [file pone.0052119.s005.doc]

Table S1. Effect of IRT5 probiotics treatment on ongoing EAMG

|  | Clinical score in 6 weeks | | | | |  |  |
| --- | --- | --- | --- | --- | --- | --- | --- |
| Treatment | 0 | 1 | 2 | >3 | | Mean Clinical Score | Δ Weight  0-6 wk(g) |
| PBS | 0/17 | 9/17 | 5/17 | 2/17 | | 1.76 | 6.8 + 12.2 |
| Pro | 5/16 | 4/16 | 2/16 | 5/16 | | 1.69 | -6.8 + 14.5 |
|  |  |  |  |  |  |  |  |
